# Primary pulmonary lymphoma in Peru

**DOI:** 10.3332/ecancer.2023.1559

**Published:** 2023-06-15

**Authors:** Elily Dianet Apumayta Requena, Danery Valdez Ocrospoma, Jhonatanael Salvador Ruiz, Alberto De la Guerra Pancorvo, Edgar Amorin Kajatt

**Affiliations:** 1 Surgical Oncology Resident, Instituto Nacional de Enfermedades Neoplásicas, Lima, Perú; 2 Oncological Pathology Fellow, Instituto Nacional de Enfermedades Neoplásicas, Lima, Perú; 3 Medical Student, San Martin de Porres University, Lima, Perú; 4 Thoracic Surgical Oncology Unit, Instituto Nacional de Enfermedades Neoplásicas, Lima, Perú; a https://orcid.org/0000-0002-1828-7009; b https://orcid.org/0000-0002-7872-7950; c https://orcid.org/0000-0002-2399-957X

**Keywords:** lymphoma, primary lung lymphoma, lung neoplasms

## Abstract

**Objective:**

To describe the clinical features, imaging, pathology and management of patients with primary pulmonary lymphoma (PPL).

**Methodology:**

This is a case series study involving a retrospective analysis of 24 patients diagnosed with PPL between the years 2000–2019 at Instituto Nacional de Enfermedades Neoplásicas in Lima, Perú.

**Results:**

73.9% of patients were male. Cough (78.3%) and weight loss (56.5%) were the most frequent clinical features. Dyspnoea and elevated values of DHL and B2 microglobulin were frequently altered in advanced stages. Diffuse large B cell lymphoma (DLBCL) represented 47.8% of the cases and the most common radiologic alterations were a mass (60%) and consolidation with air bronchogram (60%). The most utilised treatment was chemotherapy alone (60%). Three patients received only surgery. Median survival was 30 months. Five overall survival was 45%, and up to 60% in the case of mucosa-associated lymphoid tissue lymphoma.

**Conclusion:**

PPL is infrequent. Clinical features are unspecific and the principal finding is a mass, nodule or consolidation with air bronchogram. Definitive diagnosis needs biopsy and immunohistochemistry. There is no standard treatment, it depends on histology type and stage.

## Introduction

Pulmonary involvement in lymphoma can be primary or secondary. The latter is a systemic disease involving aggressive B-cell subtypes. Primary pulmonary lymphoma (PPL) accounts for 0.3% of malignant primary lung neoplasms, less than 1% of lymphomas and 3.6% of extranodal lymphomas [1, 2]. PPL is defined as a malignant monoclonal lymphoid proliferation in the pulmonary parenchyma of patients with no type of detectable extrapulmonary involvement at least 3 months after diagnosis [1].

PPL affects a similar proportion of men and women, with an average age of 55 years. Its clinical manifestations are non-specific and mainly include a cough, dyspnoea, and chest pain. On computed tomography (CT), it generally presents as a solid mass or an area of consolidation, focal or diffuse ground-glass opacities, with perilymphatic and peribronchovascular distribution [3, 4].

Definitive diagnosis requires a biopsy and immunohistochemistry. CT-guided biopsy has a sensitivity of 80%, while the sensitivity of a fibreoptic bronchoscopy with transbronchial biopsy is between 31% and 88% for mucosa-associated lymphoid tissue (MALT) lymphoma [4]. Other procedures that can aid in the diagnosis, although less common, are endobronchial ultrasound and surgical biopsy [5]. Histopathology will show interstitial infiltration of small lymphocytes forming a mass in the bronchiolar mucosa, which mainly expresses CD20+, CD5- and CD10- [4].

A broad range of treatments for PPL have been described, including observation, surgery, radiotherapy or systemic therapy, depending on the clinical stage (CS) of the disease. Systemic therapy can use a combination of agents, as the CHOP regimen (chemotherapy regimen that includes Cyclophosphamide, doxorubicin, vincristine, and prednisone) does, or a single agent, such as chlorambucil, fludarabine and rituximab. The latter has shown a response rate of up to 70% and a recurrence rate of 36% [2]. Notably, antibiotic therapy is ineffective in primary pulmonary MALT lymphoma as no associated microorganism has been identified [4].

We present a series of patients diagnosed with PPL between 2000 and 2018 at the National Cancer Institute in Lima, Peru.

## Materials and methods

A retrospective review was conducted of patients diagnosed with PPL over the last 20 years in a national referral tertiary care centre with a thoracic surgical oncology unit. All diagnoses were confirmed by anatomic pathology. The study covered 12,301 lymphoma cases over 20 years, including 23 PPL cases (0.19%), or 0.29% of 8,011 pulmonary neoplasms diagnosed during this period.

## Results

We present a series of 23 patients, 6 females and 17 males, with an average age of 58 years, and a median age of 62 years (33–77 years), with anatomic pathological confirmation of PPL. Most patients presented with a cough and weight loss as their main symptoms, with other non-specific symptoms, like chest pain and haemoptysis. Twenty-six per cent had a fever and 39% had other B symptoms associated with lymphoma. Only one CS IE patient presented with dyspnoea. A malignant neoplasm was the initial diagnostic impression in most cases (87%). However, two patients first received treatment for pneumonia and one for pulmonary tuberculosis (Table 1).

The main diagnostic procedures used were thoracotomy with pulmonary nodule biopsy and CT-guided fine needle aspiration (FNA) biopsy. Forty-four percent of cases required multiple procedures to make a definitive diagnosis. Patients with CS IIE - IV also required a fibreoptic bronchoscopy and an extrathoracic lymph node biopsy.

In the CS I group (34.8% of cases), 62.5% of patients were males, 50% had no comorbidities and the average age was 53.8 years. Two patients (25%) reported having HIV infection. Their main clinical symptoms were a cough, chest pain, weight loss and other B symptoms. A malignant neoplasm was the initial presumed diagnosis in this group (75%). The most common diagnostic procedures were FNA or Trucut biopsy and thoracotomy. Definitive diagnoses for half of the patients in this group relied on a single procedure. The median time disease had progressed before patients had a consultation was 3.5 months, and it took another month for treatment to begin.

Lugano reported 15 CS II–IV patients with an average age of 61.5 years, 80% of them males and 73% with comorbidities. Cough (80%), dyspnoea (66%) and weight loss (66%) were the most common clinical symptoms for these stages. However, only three patients had haemoptysis. The median time from symptom onset to consultation was 5 months and another month until treatment began. Lugano also described a case that for non-medical reasons took 24 months before treatment began. Nine patients (60%) only required a single diagnostic procedure.

A review of patient medical histories showed that four patients with diffuse large B cell lymphoma (DLBCL) reported having an HIV infection, one reported idiopathic thrombocytopenic purpura and another reported chronic Hepatitis B virus (HBV). Similarly, a patient with a lymphomatoid granulomatosis form of DLBCL had a malignant renal neoplasm. A human T lymphotropic virus type I (HTLV-1) infection was reported in a case of anaplastic large T-cell lymphoma. A history of asthma and other non-communicable diseases was reported in a patient with B-cell non-Hodgkin’s lymphoma (NHL). These symptoms and conditions are shown as comorbidities in Table 1.

Other diagnostic laboratory tests included the beta-2 microglobulin assay, which had a median value of 4.2 mg/L, higher than the normal range. There were no significant statistical differences between the two CS groups. Elevated levels of lactate dehydrogenase (LDH) were also found in the overall study population, with a median value of 484 IU/L. The average white blood cell (WBC) count was 8.3 × 10^9^/L, with no significant statistical differences between the two groups. Similarly, the average haemoglobin level was 11.6. (Table 2)

**Figure 1.**
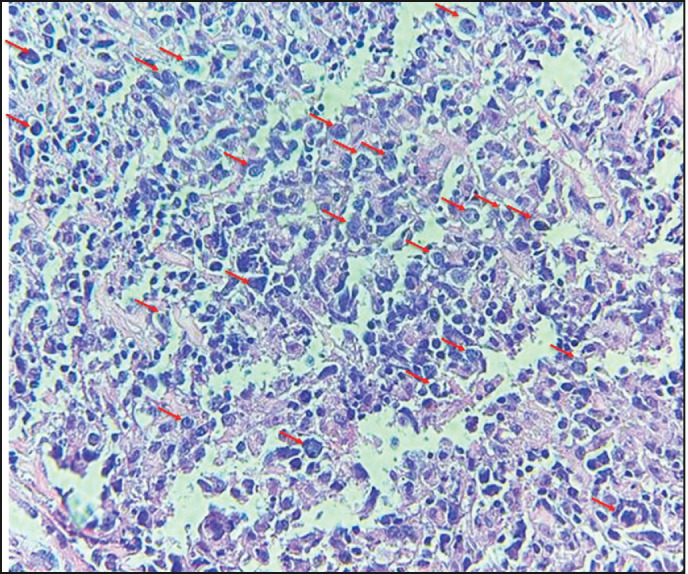
The image shows diffuse layers of large cells that destroy or replace the normal pulmonary parenchyma. Large cells resemble centroblasts and/or immunoblasts and are 2–4 times larger than normal lymphocytes, with atypical features (red arrows).

In radiological imaging (Figure 1), PPL presented mainly as a pulmonary mass (82%), and less frequently as multiple nodules and consolidation with air bronchogram. Pleural effusion also appeared in 34% of cases. Distribution in over half of cases featured peripheral involvement in the parenchyma. The largest average lesion diameter was 6.9 cm. No patient had images consistent with ground-glass opacities or signs of angiogram. Lesion distribution was peripheral in most patients with MALT lymphoma but indistinct for patients with DLBCL (Table 3).

**Figure 2.**
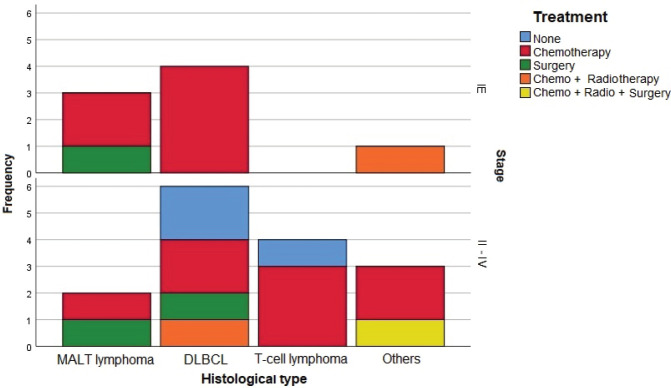
Treatment used by histological type and CS. CT: Chemotherapy. RT: Radiotherapy. CS: Clinical stage

Histological testing found the most common disease among patients was DLBCL (47.8%), followed by MALT lymphoma (21.7%). Most patients with DLBCL had advanced-stage diseases. Most with MALT lymphomas had CS I disease, while patients with T-cell lymphomas and other NHL had a disease in CS ≥ II (Figure 2).

Sixty percent of patients received chemotherapy alone as treatment. Three cases were treated with surgery alone, two in CS I and one in CS II. One case was treated with a combination of chemotherapy, radiotherapy and surgery, in a patient with CS IV B-cell lymphoma. Three cases in CS ≥ II received no treatment, two patients who died before starting a regimen and one patient who refused chemotherapy (Figure 2).

**Figure 3.**
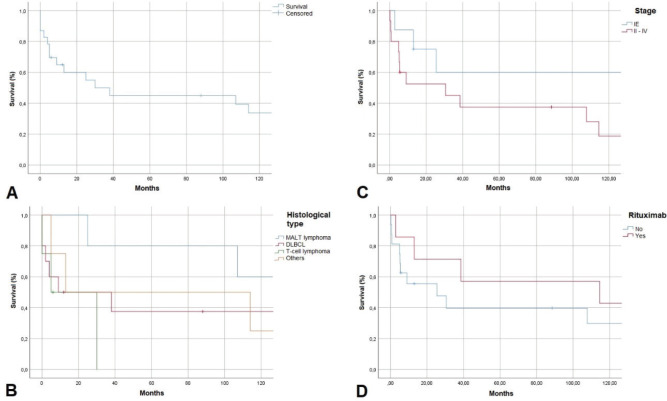
Kaplan-Meier survival curve in months. A. OS, B. OS by histological type, C: OS by clinical stage, D: OS with rituximab use

Examining follow-up and survival, we found that 2 of 23 patients were lost to follow-up, and two were followed for over 200 months. As shown in Figure 3, 1-year overall survival (OS) exceeded 60%. The average survival was 30 months. Five-year OS was 45%, a rate that persisted to 100 months, but the 10-year OS rate was 35%. MALT lymphoma had the best 5-year OS rate of all histological types, 60%. DLBCL patients survived an average of 10 months. No patient with T-cell lymphoma survived past 30 months. Examining 10-year OS rates by CS, we found a difference between patients with CS IE disease, 80%, and patients with CS II–IV disease, 20%. Examining survival in patients who received rituximab, we found that 42% in CS IE and 30% in CS II–IV survived. Our study found no cases of disease-free survival.

## Discussion

PPL represents less than 1% of all lymphomas. It is classified mainly as Hodgkin’s lymphoma (HL) or NHL [6]. The most described NHLs include MALT or MALT subtypes (42%–90%), DLBCL (24%) and T-cell lymphomas (4%) [1, 6, 7].

In our series, this ratio is reversed, with nearly half of the patients having DLBCL and less than one-fourth having MALT lymphoma. We lack a Latin American case series from which we could infer the behaviour of PPL in Peru and that would explain this distribution. However, late diagnosis (65% in CS ≥ II) may signify that those patients had a disease that had transformed into DLBCL. MALT lymphomas in our study were mainly in CS I. Conversely, more advanced CS predominated in DLBCL, with only 38% of patients were in CS I at diagnosis. There were no cases of HL.

There are several classification systems for PPL staging. Ferraro *et al* [8] have proposed a system for extranodal lymphomas that uses the Ann Arbor method. That method designates an extranodal location as *E,* and higher stage numbers to reflect the spread of the disease. Thus, CS IE signifies disease only in one or both lungs, while CS IIE signifies lymph node involvement (II1E corresponding to hilar nodes, II2E to mediastinal nodes and II2EW to the adjacent chest wall or diaphragm). CS III involves lymph nodes below the diaphragm and CS IV involves extralymphatic tissues or organs. This study used the Lugano staging system [9].

**Figure 4.**
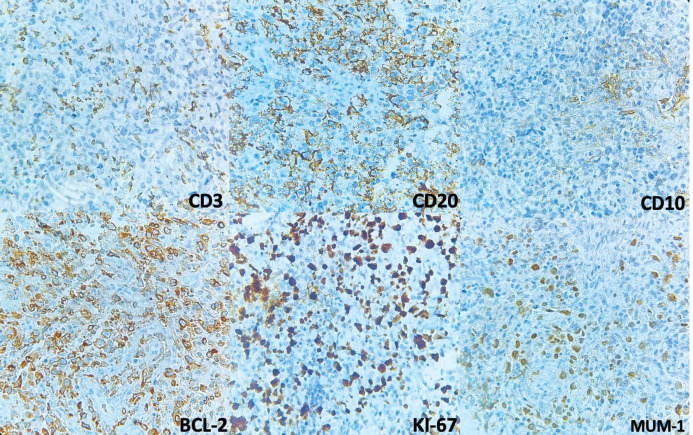
Immunohistochemistry. CD3 is negative and shows T-lymphocytes in the background. CD20 and BCL2 are positive in the large lymphocytes. KI-67 shows increased proliferation and is positive in cells with nuclei. CD10 was negative in neoplastic cells but positive in what looks like residual germinal centres. MUM-1 is positive in nuclei.

Primary MALT lymphomas of the lung are generally described as a small B-cell infiltration with lymphoepithelial lesions that appear on CT scans as single or multiple lesions with air bronchogram [7] (Figure 4). The infiltrate surrounds and sometimes colonises the reactive germinal centres. In the periphery of the lesion, neoplastic cells track bronchovascular bundles, but the centre of the lesion often shows alveolar destruction [10]. Lymphoepithelial lesions are common. Necrosis is rare and its presence should raise concern that disease is transforming into DLBCL.

One hypothesis is that extranodal MALT lymphomas develop after long-term antigenic stimulation in the context of chronic lymphoid hyperplasia. Multiple genetic subsets have been identified, including one group associated with MALT1 translocations (25%–45% of cases) and the activation of the NF-κB pathway. Another group is associated with an increase in plasma cells and a pattern of plasmacytic gene expression [11].

It is important to distinguish pulmonary MALT lymphoma from reactive lymphoid proliferations, including nodular lymphoid hyperplasia, lymphocytic interstitial pneumonia and follicular bronchitis. Aberrant CD43 expression in B-cells or plasma cells with light chain restriction can be useful, but each finding is present in only a subset of cases. Detection of clonal immunoglobulin gene rearrangement and/or translocations associated with MALT1 is useful for diagnosis in problematic cases [12].

Clinically, over half of these cases are asymptomatic and diagnosis requires transbronchial or percutaneous biopsy. MALT can be treated with surgery or radiotherapy in localised stages. We found five cases of MALT lymphoma in our series, most in patients in CS I with a cough and chest pain. Thoracotomy was superior for definitive diagnosis. Lesion distribution was mainly peripheral. Surgery controlled the disease in two patients and the other three benefitted from chemotherapy.

Our study found three cases of T/NK-cell lymphoma, in two males and one female. Their clinical manifestations included cough, fever, haemoptysis, dyspnoea and B symptoms. Radiological scans show T/NK-cell lymphoma as a pulmonary consolidation associated with hilar adenopathy and pleural effusion. It is, therefore, often confused with pneumonic processes [13], all in CS IIE. One of these three patients reported HTLV-1 infection. But the possibility of secondary lung infiltration was ruled out in the context of a possible anaplastic T-cell lymphoma suggested by tests that did not show disease spread. One T/NK-cell lymphoma patient died before starting chemotherapy. Another died of acute renal failure after receiving one course of CHOP. The third patient died after receiving three courses of the GELOX regimen (chemotherapy regimen that consisted of combined gemcitabine, oxaliplatin, and L-asparaginase).

DLBCL predominated in our series, but usually represents 10%–19% of PPL. It can result from the aggressive transformation of MALT lymphoma, mainly when associated with HIV infection, collagen diseases or immunosuppressive states. Unlike previously described lymphomas, DLBCL can also appear on radiological scans as cavitation and/or areas of necrosis. Ten-year survival is currently 90% [14]. Radiological manifestations of DLBCL vary, but it is mainly characterised by parenchymal masses over 5 cm in diameter. Morphologically, all cases showed diffuse layers of large cells replacing normal pulmonary parenchyma. Most DLBCL patients were not tested for bovine enterovirus, which is relevant to this kind of lymphoma, especially in immunocompromised patients. Several DLBCL treatments were used, including surgery alone, but the main treatment was the CHOP chemotherapy regimen. Among the comorbidities described, three patients had HIV infection and one had immune thrombocytopenic purpura.

No optimal treatment for PPL has been established. Surgery, with or without adjuvant chemotherapy, or chemotherapy alone has been described, and surgery is not contraindicated in cases with hilar or mediastinal lymph node involvement. Complete resection with regional lymph node dissection is an option up to CS IIE. Adjuvant chemotherapy following an R0 resection, preferably for high-grade NHL, remains controversial [15, 16]. Four cases in our study featured surgery to control disease, one with adjuvant chemotherapy and radiotherapy for CS II disease.

The most used chemotherapy we found was the CHOP regimen (65%). Half of patients who received CHOP had CD20 expression and also received rituximab. Follow-up treatment using the ESHAP regimen (chemotherapy regimen that consists of combined etoposide, methylprednisolone, high-dose cytarabine, and cisplatin) was required for one case of CS IIE lymphomatoid granulomatosis B-cell lymphoma and two cases of DLBCL in CS IE and IIE. The cyclophosphamide, vincristine sulfphate, and prednisone regimen was first used in one case of CS IV MALT and one case of CS IV B-cell NHL IV, followed by CHOP in both cases and the ifosfamide, carboplatin, and etoposide regimen in the second. One patient received the GELOX regimen for CS IIE T-cell lymphoma. A patient with CS I mantle lymphoma received the R-CHOP and R-DHAP regimens (rituximab, dexamethasone, cisplatin and cytarabine). A patient with CS IE MALT lymphoma received the fludarabine, mitoxantrone, and dexamethasone regimen .

Various prognostic factors have been studied, such as bilateral involvement, presence of metastases, surgery, use of adjuvant chemotherapy, and high levels of B2 microglobulin and LDH [17, 18]. Among the variables we studied, dyspnoea was associated with PPL in CS ≥ II at diagnosis. There was no statistically significant difference, but the average time to disease appearance was higher for CS ≥ II, 5 versus 3.5 months for CS I. LDH and B2 microglobulin (*p* > 0.05 for both) were higher for CS ≥ II. LDH was 491 in CS ≥ II versus 394 IU/L in CS I. Elevated B2 microglobulin values have been associated with higher potential for invasiveness and higher tumour load [17]. Our study found higher levels of this protein in CS ≥ II, 3.1 ug/dL versus 1.4 in CS I.

The most common useful diagnostic techniques are CT-guided percutaneous biopsy, flexible fibreoptic bronchoscopy and surgery [19]. The choice of diagnostic technique depends on disease presentation and the availability of those techniques. The usefulness of 18-fluorodeoxyglucose positron emission tomography (PET)/CT has been shown, and this technique makes it possible to distinguish PPL by its metabolism and morphology. It also helps determine disease staging and biopsy site selection [20]. However, access to this technology is still limited in our area.

**Figure 5.**
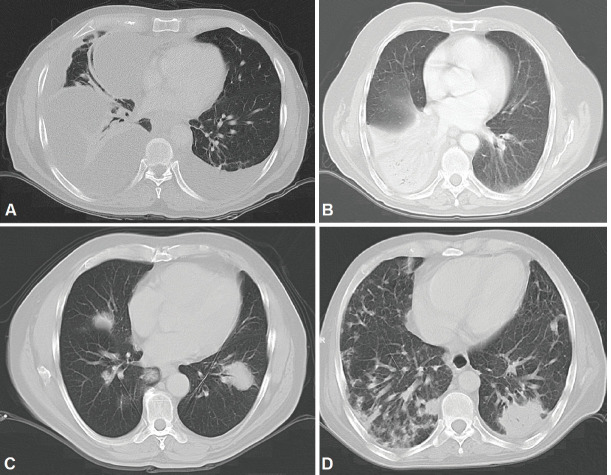
CT scans. A: Bilateral pleural effusion mainly right, loculated pleural effusion B. Consolidation with left lower lobe air bronchogram. C: Consolidation with air bronchogram D: Bilateral multifocal consolidations with air bronchogram.

Radiologically, PPL has been described as an area of single consolidation or multiple parenchymal nodules, and less frequently as a consolidation-type lesion with air bronchogram, cavitations or single nodules [21]. Radiological images of MALT lymphoma have shown peripheral or diffuse distribution of mass lesions, consolidation or nodules (Figure 5). DLBCL has presented lesions with a similar frequency, but with central distribution in slightly less than half of cases and associated with regional adenopathies in 63.6% of cases (Figure 1). We, therefore, used FNA or Trucut biopsies, thoracotomy plus biopsy, and fibreoptic bronchoscopy with our patients. In over half of cases, a single method was sufficient to reach a definitive diagnosis. Our main differential PPL diagnoses included pulmonary plasma cell granuloma, Wegener’s granulomatosis, inflammatory pseudotumour, sarcoidosis, intrapulmonary adenopathies, Castleman disease and other conditions.

A multidisciplinary review is especially valuable in diagnosing haematolymphoid tumours. For example, CT scans showing specific characteristics of MALT lymphoma may help exclude non-MALT lymphomas and reactive disorders such as lymphoid interstitial pneumonia [22]. Moreover, a joint review by thoracic and haematolymphoid pathologists can be valuable as both subspecialties can bring unique expertise to the diagnostic process.

OS rates of 57% at 3 years and 46% at 5 years have been reported. Progression-free survival (PFS) is 53% at 3 years and 44% at 5 years. Average OS and PFS are 52 and 45 months, respectively [6]. We did not examine PFS but found 3 and 5-year OS rates were fairly similar to the established respective rates of 50% and 45%. Figure 3 shows lower survival rates for higher CS and the influence of rituximab use in increasing the survival of patients with lymphoma.

Our study has the following limitations. First, data were obtained retrospectively by reviewing clinical records. However, information was systematically collected and checked, and pathology findings were re-reviewed to verify they were representative. No routine PET/CT scans were performed due to limited access to this technology in our area.

## Conclusion

PPL is rare. DLBCL is the most common lymphoma in our area, with most patients in advanced CS at diagnosis. The next most common lymphoma is MALT, mainly CS I cases with non-specific clinical manifestations such as cough, chest pain, weight loss and, for patients in stages II or higher, dyspnoea. LDH and B2 microglobulin values are usually elevated, mainly in CS II. Biopsies and immunohistochemistry are essential for a definitive diagnosis. The biopsy technique depends on the type and distribution of the lesion. The disease is mainly found as a mass, nodule or consolidation with an air bronchogram, either singly or as multiple lesions. There is no standard treatment protocol, but most patients benefit from chemotherapy and surgery is useful in localised disease. Some patients require multiple modes of treatment, including radiotherapy, depending on the extent of their disease.

## Conflicts of interest

The authors declare that they have no conflicts of interest.

## Funding

The authors have not received any kind of funding for the research and writing of this paper.

## Figures and Tables

**Table 1.** Clinical-epidemiological characteristics of patients with PPL.

Variable	Total (*n* = 23)	CS I (*n* = 8)	CS II-IV (*n* = 15)	*p*-value^a^
Age, years ± SD	58.8 ± 13.2	53.8 ± 16.4	61.5 ± 10.9	0.190
Male, *n* (%)	17 (73.9)	5 (62.5)	12 (80)	0.363
Comorbidities, *n* (%)	8 (34.8)	4 (50)	11 (73.3)	0.263
Clinical manifestations, *n* (%)				
Cough	18 (78.3)	6 (75)	12 (80)	0.782
Dyspnoea	11 (47.8)	1 (12.5)	10 (66.7)	0.013
Chest pain	10 (43.5)	4 (50)	6 (40)	0.645
Haemoptysis	4 (17.4)	1 (12.5)	3 (20)	0.651
Fever	6 (26.1)	1 (12.5)	5 (33.3)	0.278
Weight loss	13 (56.5)	3 (37.5)	10 (66.7)	0.179
Other B symptoms	9 (39.1)	2 (25)	7 (46.7)	0.311
Initial MN diagnosis, *n* (%)	20 (87.0)	6 (75)	14 (93.3)	0.214
T. Disease, months (range)	4 (1–12)	3.5 (1–7)	5 (1–12)	0.494
Time to treatment, months (range)	1 (1–24)	1 (1–12)	1 (1–24)	0.943
Diagnostic procedure, *n* (%*)*				0.360
VATS	1 (4.3)	1 (12.5)	0 (0)	
Thoracotomy	7 (30.4)	3 (37.5)	4 (26.7)	
FNA or Trucut biopsy	7 (30.4)	3 (37.5)	4 (26.7)	
Fibreoptic bronchoscopy	5 (21.7)	1 (12.5)	4 (26.7)	
Lymph node biopsy	3 (13)	0 (0)	3 (20)	
Single diagnostic procedure, *n* (%)	13 (56.5)	4 (50)	9 (60)	0.645

a Student *T*-test or Mann-Whitney U Test for continuous variables; Chi-square test for categorical variables

**Table 2.** Ancillary laboratory tests.

Variable	Total (*n* = 23)	CS I (*n* = 8)	CS II-IV (*n* = 15)	*p*-value^a^
LDH, IU/L (range)	484 (281–1,627)	394 (342–1,013)	491 (281–1,627)	0.549
Beta-2 microglobulin, ug/mL (range)	4.2 (1.2–20.6)	1.39 (1.8–18.7)	3.1 (1.2–20.6)	0.589
WBC × 10 9/L ± SD	8.3 ± 4.9	8.1 ± 4.4	8.4 ± 5.4	0.896
Haemoglobin, g/dL ± SD	11.6 ± 2.9	11.1 ± 2.4	11.9 ± 3.2	0.557

a Student *T*-test or Mann-Whitney U test for continuous variables; Chi-square test for categorical variables

**Table 3.** Radiological characteristics on CT scans in patients with PPL.

Variable	MALT (*n* = 5)	DLBCL (*n* = 10)	Other (*n* = 8)	*p* ^a^
Largest diameter, cm ± SD	5.4 ± 2.3	6.7 ± 4.4	9 ± 4.8	
Distribution, *n* (%)				0.347
Central	0	5 (50)	4 (50)	
Peripheral	4 (80)	5 (50)	4 (50)	
Diffuse	1 (20)	0	0	
Regional adenopathies, *n* (%)	1 (20)	6 (60)	5 (62.5)	0.367
CT finding, *n* (%)				
Mass	3 (60)	8 (80)	8 (100)	0.319
Consolidation and air bronchogram	3 (60)	2 (20)	1 (12.5)	0.205
Multiple nodules	2 (40)	2 (20)	3 (37.5)	0.101
Pleural effusion	1 (20)	2 (20)	5 (62.5)	0.028
Pleural thickening	0	2 (20)	1 (12.5)	0.516

a Chi-squared test for categorical variables
